# Impact of Climate Variation on Land Use Land Cover Change in Kassala State, Sudan

**DOI:** 10.1155/tswj/9005930

**Published:** 2025-12-26

**Authors:** Awad Elkarim Suliman Osman Khalifa, Hassan Elnour Adam, Faisal Ismail Musa

**Affiliations:** ^1^ Institute of Gum Arabic Research and Desertification studies, University of Kordofan, El Obeid, Sudan, kordofan.edu.sd; ^2^ Department of Forestry, University of Kordofan, El Obeid, Sudan, kordofan.edu.sd; ^3^ CEN-Center for Earth System Research and Sustainability, University of Hamburg, Hamburg, Germany, uni-hamburg.de; ^4^ Department of Forestry, University of Blue Nile, Ad-Damzin, Sudan; ^5^ Department of Forestry, Mizoram University, Aizawl, India, mzu.edu.in

**Keywords:** climate variation, Kassala state, land use land cover, windstorms

## Abstract

Change in global weather pattern has become a major concern because of their direct impact on land use and land cover (LULC). This study, conducted in Kassala State, Sudan, examines how climatic variability influences these changes. Additionally, the study is intended to find out the possible intervention to reduce the impact of wind and sandstorms in the area. Primary data were collected through interview, focus group discussion, direct observation, and analysis of satellite images. Landsat TM 5 (2002) and Landsat 8 OLI (2023) images were used for LULC mapping. Meteorological data were obtained from local weather stations, while social data were gathered through stakeholder interviews. Quantitative data were analyzed using SPSS and Microsoft Excel while satellite images were processed using supervised classification and change detection techniques in ERDAS and ArcMap. The result showed fluctuations in rainfall (*R* ^2^ = 0.04), an increase temperature (*R* ^2^ = 0.01), and higher wind speeds (*R* ^2^ = 0.02). Forest cover declined from 17.11% to 13.20%, while bare land and shrubland expanded. The study recommends agroforestry systems interventions including windbreak and shelterbelts using *Acacia tortilis* and *Acacia raddiana* to mitigate the effects of sandstorms and land degradation.

## 1. Introduction

Sudan is highly vulnerable to the impact of climate change because of its arid to semi‐arid conditions and dependence on rain‐fed agriculture [1–3]. Rainfall fluctuation and temperature rising lead to change in agriculture pattern and land use land cover [3, 4]. However, the situation is worse by prevailing conditions of poverty and other environmental factors that create several pressing challenges for Sudan [5–7]. Agriculture is inherently sensitive and directly affected by climate variability and is one of the most vulnerable sectors to the risks and impacts of global climate change [8–10]. Climate variation is considered as the greatest threat to agriculture production and food security in agriculture‐based countries [2], because of their low adaptive capacity to effectively cope with a possible reduction in yields among others [11]. Conversely, several studies predict rising temperature and declining rainfall [3, 12]. Thus, the variations and erratic nature of rainfall composed with its concentration during the short growing season increases the exposure of the rain‐fed agricultural system [13]. Additionally, adverse climate variations such as variation in temporal and spatial rainfall determine the density and composition of the vegetation cover [14, 15]. Although an increase in global temperatures may extend the plants growing season [16], meanwhile, an increased temperature of 1°C–2°C could negatively impact crops growth and production in low‐latitude regions [17].

Climate change is expected to have a significant impact in all ecosystem level of terrestrial biodiversity and genetic diversity [18]. However, changes in climate factors will excite changes in species diversity, range, life cycle and behavior, as well as genetic development responses over time [19, 20]. Simultaneously, a decline in genetic variety and a threat to pollination facilities are the results of changes in species fitness in response to climate fluctuation, as indicated by changes in species abundance, distribution, and phenology [21, 22]. Reduced habitat and other human‐induced pressure threaten up to 50% of Africa’s overall biodiversity [23, 24]. Additionally, the advancement of desertification will accelerate because of rising temperatures and changes in other climatic conditions [25–28].

Kassala State, located in eastern part of Sudan, is among the most affected region because of the frequent droughts, sandstorms, and advancing desertification. The area supports mixed livelihoods agriculture, pastoralism, and small‐scale trade yet land productivity has declined because of rainfall variability and vegetation loss. Previous studies have examined climate variation and land cover changes in other parts of Sudan, such as North Kordofan and central regions [3, 12]. However, there is a clear knowledge gap regarding the eastern region, particularly Kassala State, which experiences frequent sand and windstorms. Understanding how climatic variations have historically shaped LULC in Kassala is critical for sustainable land management and climate adaptation. For instance, prolonged dry seasons and increasing wind intensity have accelerated the expansion of Karab (undulating) lands, while declining rainfall has limited natural regeneration of acacia‐dominated woodlands that provide fuelwood, fodder, and gum Arabic. Preliminary field observations and stakeholder consultations in Kassala confirm visible signs of forest degradation and shifting cultivation patterns, suggesting that climatic stress is a key driver of landscape change. Therefore, this study contributes new insights into the relationship between climatic variability and land use/land cover changes in Kassala State, filling a geographical and methodological gap by integrating meteorological, remote sensing, and community‐based survey. Specifically, this study aims (a) to assess the impact of climatic variation on land use and land cover change, and (b) to identify possible interventions to reduce the effects of wind and sandstorms in Kassala State. The findings contribute to understanding regional climate impacts and provide policy insights for the land management, food security, and desertification control in eastern part of Sudan.

## 2. Material and Method

### 2.1. Study Area

**Figure 1:**
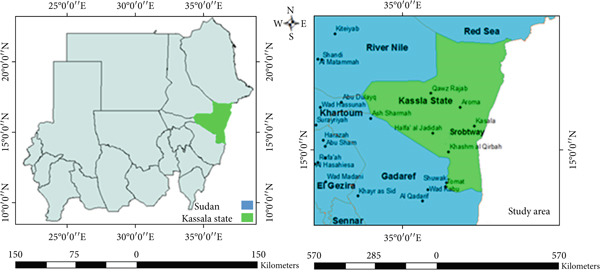
Study area.

Kassala State lies between latitudes 14° and 17° N and longitudes 34° and 37° E, and accommodating around 2.4 M population distributing over an area of 54,066 km^2^ in 11 localities (Figure 1). In Kassala State, the total arable land is around 2.8 million feddan and about 850,000 feddan representing cultivated land. The state is one of the animal‐rich states in Sudan, providing grazing land for an estimated 4 million heads of livestock and encompassing approximately 7 million feddans of pastureland [29]. Agriculture is the main practice in the area including growing crops, such as cereals, oilseeds, cotton, and peanuts (groundnuts). In the southern and northern parts of Kassala, cattle and camels are being raised [30]. Moreover, there are some industries established in the Kassala state that include spinning mills, sugar refineries, cotton ginning, soap factories, and oilseed mills. In addition to that, Kassala State is an important agricultural center and the source of border‐trade for Sudan.

### 2.2. Data Collection

Data was collected via focus group discussion including village leader, forest national corporation (FNC) office, ministry of production, and economical resources, direct interview as well as direct observation. In addition, a total of 77 respondents were randomly selected to participate in the questionnaire survey conducted to capture community perceptions on climate variability and its impacts on land use and vegetation cover. However, social data were supported by remote sensing data, where satellite imagery covering the study area were downloaded, including Landsat 5 Thematic Mapper (TM dated 01.02.2002) and Landsat 8 OLI_TIRS (ETM+ dated 23.02.2023). The two cloud free Landsat images cover a scene with path 191 and row 49, which included the study area. However, these images were acquired during the dry season and identified some characters as shown in Table 1. Landsat 5 (2002) and Landsat 8 (2023) were selected for their 30‐m spatial resolution, ensuring accurate LULC comparison. The classification accuracy was evaluated using 20 ground control points and a confusion matrix, achieving an overall accuracy of 87% and a kappa coefficient of 0.82. Moreover, rainfall, temperature, and wind speed for 10 years were collected from the metrological stations in Kassala State.

**Table 1:** Characteristics of Landsat 5 and Landsat 8 images.

**Landsat 5 (TM)**			**Landsat 8**		
**Bands**	***λ* (m)**	**R (m)**	**Bands**	***λ* (m)**	**R (m)**
1 Blue	0.45–0.52	30 m	1Coastal/Aerosol	0.435–0.451	30 m
2 Green	0.52–0.60	30 m	2 Blue	0.45–0.512	30 m
3 Red	0.63–0.69	30 m	3 Green	0.53–0.512	30 m
4 NIR	0.76–0.90	30 m	4 Red	0.636–0.67	30 m
5 SWIR1	1.55–1.75	30 m	5 NIR	0.85–0.879	30 m
6 TIR	10.40–12.5	120 m	6SWIR1	1.566–1.65	30 m
7 SWIR2	2.08–2.35	30 m	10 TIR1	10.6–11.19	100 m
			11 TIR2	11.5–12.51	100 m
			7 SWIR2	2.10–2.294	30 m
			8 Pan	0.50–0.676	15 m
			9 Cirrus	30 m	30 m

*Note:* R = spatial resolution, *λ* band length.

### 2.3. Data Analysis

Social and meteorological datasets were further analyzed in SPSS (Version 22) to compute descriptive statistics (percentage) and test correlation between climatic variables such as rainfall, temperature, and wind speed. In the meantime, Origin pro (2025) and Excel were used for graph creation. In contrast, standardized precipitation index was calculated using equation, which developed by Adam [31] following Formula (1). While remote sensing data, satellite imagery was analyzed qualitatively via visual interpretation and quantitatively using supervised classification (maximum likelihood classification) as described by McKee et al. [32]. However, to identify changes in different land cover in the area, change detection was adopted using spectral pattern recognition method. Change detection procedure following the method described by Franklin [33]. All satellite imagery were analyzed using ERDAS and ArcMap software. Nevertheless, training samples for each information class were chosen using visual perception of the pictures backed by field measurement in order to perform supervised classification. Water, bare, forest and Karab lands, (undulating lands), shrubs lands including *Prosopis chilensis* (Maskeits) and acacias, and sandy soil classes are the six classes into which supervised classification separated.

(1) SPI=P−μ/σ

Where *P* represents annual precipitation, *μ* is the mean precipitation, and *σ* is the standard deviation of precipitation.

## 3. Results and Discussion

### 3.1. Climatic Variability During Study Period

**Figure 2:**
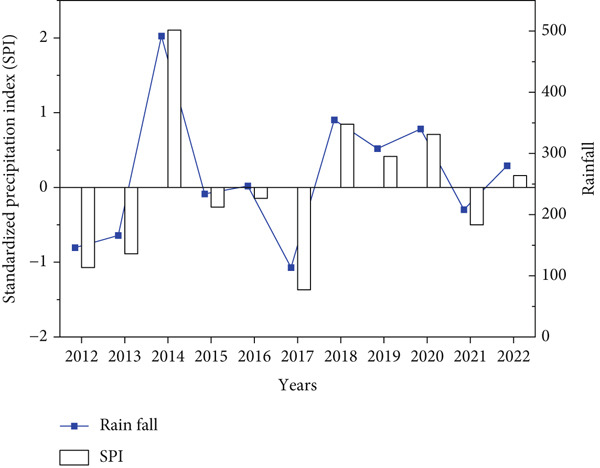
Rainfall and standardized precipitation index during study period.

**Figure 3:**
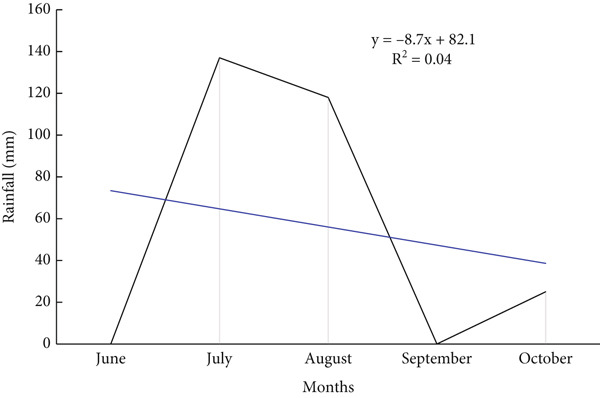
Monthly rainfall distribution in the area.

**Figure 4:**
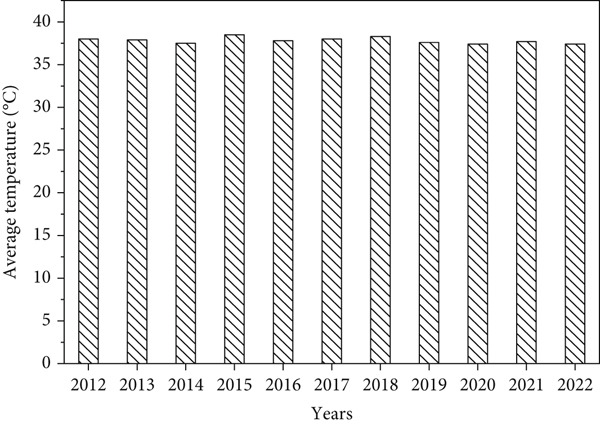
Mean annual temperature during study period.

**Figure 5:**
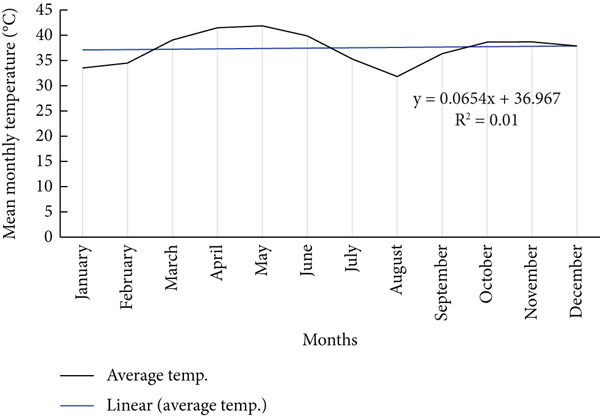
Mean monthly temperature in the area.

**Figure 6:**
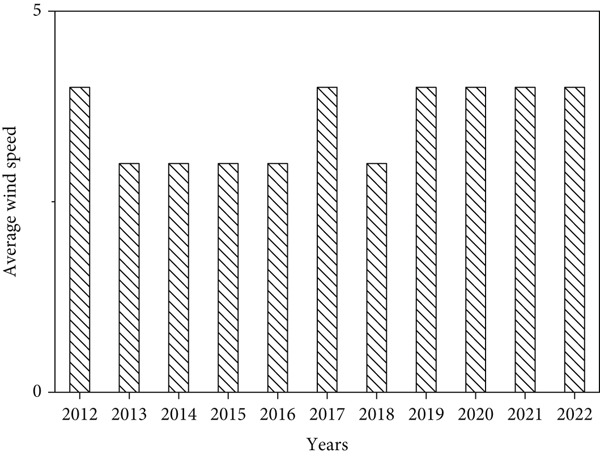
Mean annual wind speed.

**Figure 7:**
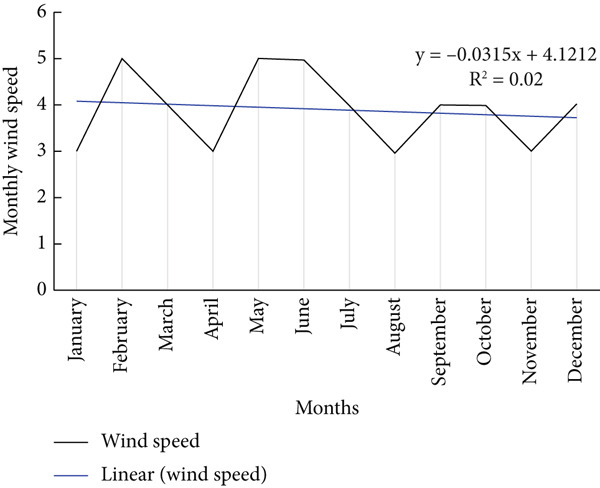
Mean monthly wind speed in the area.

**Figure 8:**
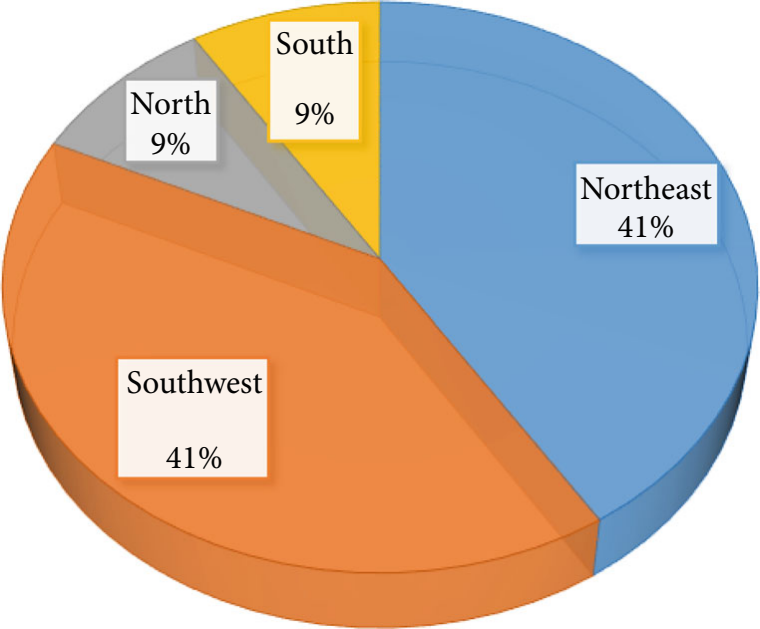
Wind direction in area through the year.

**Figure 9:**
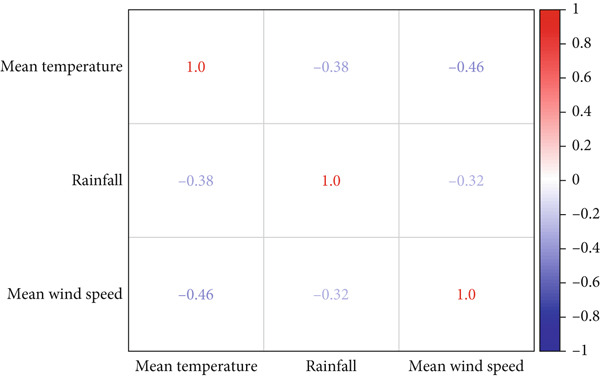
Correlation between climatic factors.

The results found that the annual rainfall varies among years during study period and clearly indicated in standardized precipitation index with an average and standard deviation of (262.69 mm/year ± 108.95) (Figure 2), while the seasonal average rainfall distribution in the area shows unequal during rainy months with *R* ^2^ = 0.04 (Figure 3); however, the area received regular rainfall in July and August while facing rainfall fluctuation in June, September, and October in each year. Similarly, Mohamed et al. [12] observed that during July, August, and September, most of Sudan states faced rainfall stability compared with other months of the year. This situation can negatively affect the vegetation cover, agricultural lands, biodiversity as well as the ecosystem in the study area. These findings are consistent with [10, 11] who noted that the fluctuation in rainfall led to vegetation cover degradation, decline in agricultural production, and substantial biodiversity losses. In addition, the results showed that the average temperature remains stable during the study period except some years that increased with an average of 37.82°C (Figure 4). While the mean monthly temperature increases from March to June and remained stable during rainy season (Figure 5). Increasing temperature may result in reduction of agricultural crop production. Similarly, Challinor et al. [17] also reported that increased temperature has a negative influence on crop growth and yield. The average annual and seasonal wind velocity increased primarily during the dry season (Figures 6 and 7), with the dominant and most damaging wind coming from northeast and southwest (Figure 8). These findings are in line with [25, 34] who stated that climate variability will increase desertification and causes serious soil erosion as the result of increasing wind speed in the region. Moreover, a negative correlation was observed between climatic factors (Figure 9). Conversely, insights from a group discussion revealed that the village head corroborated these findings, stating that crop production in the area has declined as a result of increasing temperature and reduced precipitation. In contrast, the regression coefficients (*R* ^2^ values) were relatively low; this reflects the inherent variability of climatic condition in semi‐arid regions such as Kassala. Weak linear trends are common in such environments where interannual fluctuations are driven by erratic rainfall and temperature patterns [2, 12].

### 3.2. Respondents Perception on Climate Variability

Figures 10a, 10b, 10c, and 10d present respondents’ perception of change in rainfall, temperature, wind speed, and sandstorms over the past two decades in Kassala State. However, most respondents perceived a decrease in rainfall and shorter rainy seasons (Figure 10a), which closely aligns with the meteorological data showing fluctuating and slightly declining rainfall trends (*R* ^2^ = 0.03). This perceived reduction reflects the community’s observation of delayed onset and early cessation of rains, affecting crop production and rangeland condition. In the contrast, the respondents in the area reported a noticeable increase in temperature (Figure 10a), consistent with instrumental data showing a gradual rise in mean annual and seasonal temperature particularly during summer months. Respondents attributed this warming to prolonged dry spells and declining soil moisture. Similarly, based on local respondents’ perception, increases in wind speed significantly affect crop production, sand accumulation, and houses destruction (Figure 10b), which correspond with measured data indicating a rise in wind velocity and greater variability during the dry season.

Figure 10Respondents’ perception on climate variability in the area.

**Figure:**
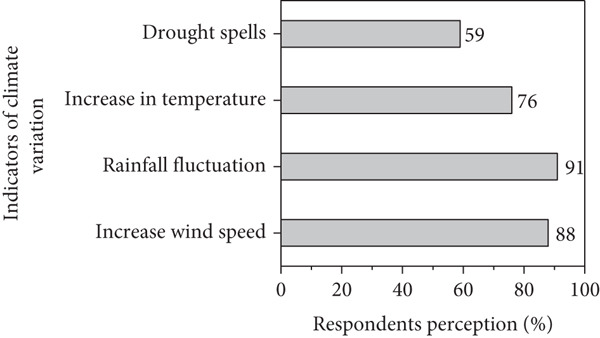
(a)

**Figure:**
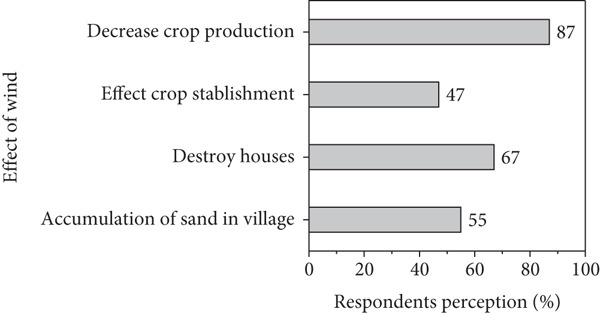
(b)

**Figure:**
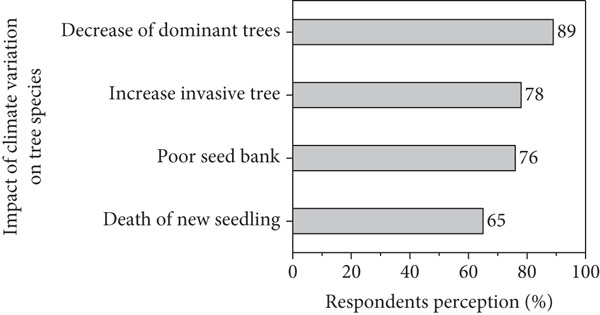
(c)

**Figure:**
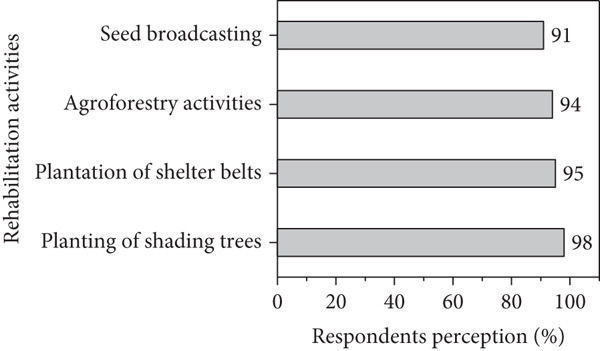
(d)

In addition, majority of respondents in the area stated that climatic variability significantly impacts new seedling, increasing invasive species while decreasing the dominant tree species in the area (Figure 10c). In contrast, the local people started using adaptive strategies such as agroforestry activities and plantation (Figure 10d) to reduce wind speed and sandstorm in the area.

### 3.3. Remote Sensing Data

**Figure 11:**
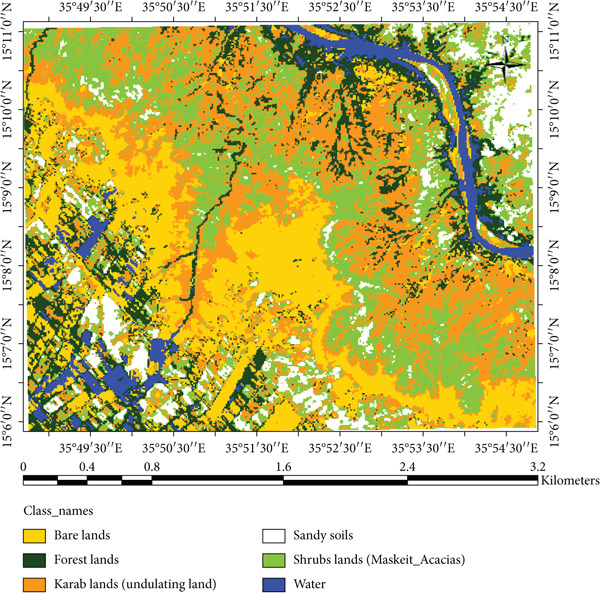
Map of classified image for Landsat 8 dated 2023.

**Figure 12:**
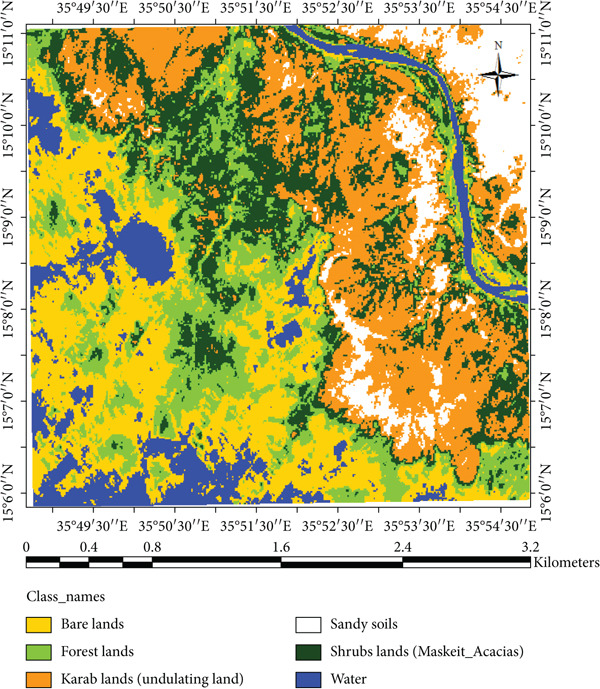
Map of classified image for Landsat 5 TM dated 2002.

**Figure 13:**
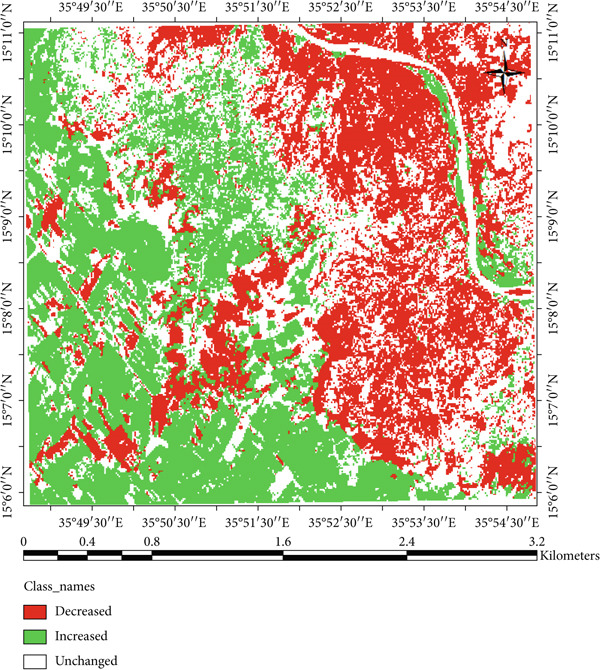
Change detection of LULC change in area between 2002 and 2023.

The result indicated that forestlands decreased from 17.11% in 2002 to 13.20% in 2023, while the Karab lands increased from 23.08% to 29.86%. The conversion of forest and shrubland into Karab and agricultural lands illustrates the combined effects of climatic stress and human pressure. In contrast, local interviews revealed that declining rainfall and prolonged dry spells have forced communities to expand cultivation into marginal lands, accelerating deforestation and soil exposure. The decrease in forests cover could be due to fluctuation in rainfall and temperature. These findings are consistent with observations by Khalifa et al. [3] and Abdi et al. [7], who observed similar vegetation decline in North Kordofan linked to climate stress and unsustainable land management. However, Kassala’s proximity to the Red Sea Hills and its exposure to strong seasonal winds make its degradation pattern distinct, as erosion and sand deposition occur more rapidly than in western Sudan. In the meantime, Musa et al. [35] and Mohammed et al. [36] reported that the increase of anthropogenic disturbance and fluctuations in climate variability significantly impact vegetation cover as well as decrease species diversity in the area. The expansion of agricultural and bare land is also observed covering large areas in 2023 (Figure 11), which reduced and affected the availability of rangelands for the livestock in the area. Conversely, these changes have been observed during the field observations and were confirmed in Figures 12 and 13. Moreover, agriculture expansion is one of the main reasons that led to the decrease of vegetation cover in the area. A huge spread of *Prosopis chilensis* trees was seen dominating the endogenous species, as similarly reported by FNC reports and Hegland et al. [21]. Eltahir et al. [37, 38] observed the demand on cash crops, which significantly lead to expansion of agriculture area and decrease in forest cover. Meanwhile, the decline in soil nutrient led to decrease in agriculture productivity. Therefore, the shrubs vegetation class, comprising *P. chilensis* and several Acacia species, expanded from 19.24% in 2002 to 23.46% in 2023. The Karab land that refers to an undulating site is covered with some endangered species, and recently also invaded by *P. chilensis* trees. This class increased by 4.22% during the study period, showing the presence of *P. chilensis* trees as new species in the area (Table 2). However, we observed changes in the LULC during the periods (2002–2023) (Table 2), resulting in the deterioration of the ecosystems that negatively influenced people’s life in the area as confirmed by communities and leaders.

**Table 2:** Distribution of LULC in study area in 2002 and 2023.

**Class name**	**2002**		**2023**		**Change %**
	**Area, ha**	**%**	**Area, ha**	**%**	
Water	1042.47	9.92	483.48	4.60	−5.32
Bare lands	2357.01	22.42	2253.6	21.44	−0.98
Forest lands	1798.38	17.11	1387.44	13.20	−3.91
Karab lands (undulating lands)	2426.85	23.09	3138.66	29.86	6.77
Shrubs lands (Maskeits + Acacias)	2022.84	19.24	2466.09	23.46	4.22
Sandy soils	864.45	8.22	782.73	7.45	−0.78

These findings emphasize the importance of integrating adaptive land management practices to mitigate wind erosion and vegetation loss. Agroforestry systems incorporating *A. tortilis*, *A. raddiana*, and *A. senegal* can serve as effective shelterbelts and windbreaks, similar to successful application in semi‐arid Sudan [3], and the Sahel [26, 27]. Such interventions improve the soil stability and ecosystem resilience under increasing climate variability. The change detection analysis (Table 3) indicates notable LULC transitions in Kassala State from 2002 to 2023. About 31.6% of the area showed a decrease in land cover, mainly forest and water bodies while 32.88% increased, particularly in Karab and shrub lands, and 36.07% remained unchanged. These shifts reflect an overall trend of vegetation degradation and landscape transformation driven by both human activities and climatic variability. Similarly, Hammad et al. [39] reported that crop production and forest cover significantly decline because of land use change. The decline in forest and water classes corresponds with decreasing and erratic rainfall pattern and rising temperatures, while the increase in shrub and Karab lands reflects encroachment of drought‐tolerant and invasive species such as *P. chilensis*. Similar patterns have been reported in semi‐arid region of Sudan and the Sahel [3, 26, 27], suggesting that continued climatic stress and land‐use pressure are accelerating desertification and highlighting the urgent need for adaptive management such as agroforestry and reforestation measures.

**Table 3:** Change detection of LULC in study area.

**Change**	**Area, ha**	**%**
Decreased	3285	31.06
Unchanged	3815.28	36.07
Increased	3477.42	32.88

## 4. Conclusion and Recommendations

This study demonstrated that the climate variability, particularly rainfall fluctuation and temperature rise, has significantly altered land use vegetation cover in Kassala State over the past two decades. Forest and rangeland degradation were accompanied by agricultural expansion and the spread of *P. chilensis*. These findings add to the understanding of climate land cover interactions in eastern Sudan, a region that has been underrepresented in previous studies. The study recommends practical measures such as agroforestry (including shelterbelts and windbreaks), afforestation, and the use of indigenous tree species such as *A. tortilis*, *A. raddiana*, and *A. senegal* that can mitigate sandstorms and enhance ecosystem resilience. Policymakers should impose land‐use planning and community‐based restoration to ensure long‐term sustainability under changing climate conditions.

## Conflicts of Interest

The authors declare no conflicts of interest.

## Funding

No funding was received for this manuscript.

## Data Availability

All data used/analyzed in this paper are available with the corresponding author and will be shared on a reasonable request.
